# Supplementary material for: Hydrogel-Assisted 3D Volumetric Hotspot for Sensitive Detection by Surface-Enhanced Raman Spectroscopy
Source: Int J Mol Sci. 2022 Jan 17;23(2):1004. doi: 10.3390/ijms23021004 (PMC8779965; doi:10.3390/ijms23021004)
Supplement: Supplementary file 1 [file ijms-23-01004-s001.zip › ijms-1515810-supplementary.pdf]

International Journal of *Molecular Sciences*

MDPI

Supplementary Material

Hydrogel-assisted 3D volumetric hotspot for sensitive detection by surface-enhanced Raman spectroscopy

Soo Hyun Lee [1], Sunho Kim [2], Jun-Yeong Yang [1], ChaeWon Mun [1], Seunghun Lee [1], Shin-Hyun Kim [2,*] and Sung-Gyu Park [1,*]

[1] Department of Nano-Bio Convergence, Korea Institute of Materials Science, 797 Changwondae-ro, Changwon 51508, Korea; sh_lee@kims.re.kr (S.H.L.); yjy8184@kims.re.kr (J.-Y.Y.); apple1025@kims.re.kr (C.M.); seunghun@kims.re.kr (S.L.)

[2] Department of Chemical and Biomolecular Engineering, Korea Advanced Institute of Science and Technology (KAIST), Daejeon 34141, Korea; shkim1020@kaist.ac.kr (S.K.)

* Correspondence: kim.sh@kaist.ac.kr (S.-H.K.) and sgpark@kims.re.kr (S.-G.P.)

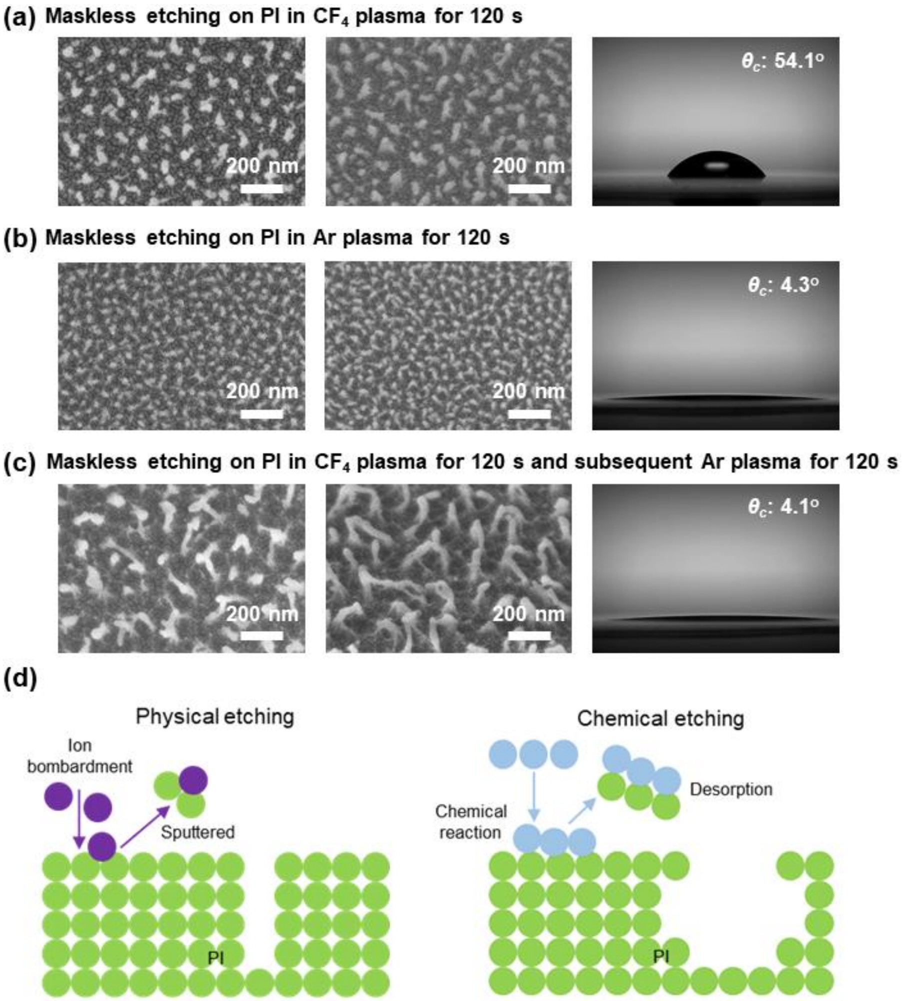

Figure S1. FE-SEM images and contact angle of PNs etched in (**a**) CF_4 plasma for 120 s, (**b**) Ar plasma for 120 s, and (**c**) CF_4 plasma for 120 s, followed by Ar plasma for 120 s. (**d**) Schematic showing the fundamentals of plasma etching.
